# Wild type and gain of function mutant TP53 can regulate the sensitivity of pancreatic cancer cells to chemotherapeutic drugs, EGFR/Ras/Raf/MEK, and PI3K/mTORC1/GSK-3 pathway inhibitors, nutraceuticals and alter metabolic properties

**DOI:** 10.18632/aging.204038

**Published:** 2022-04-27

**Authors:** James A. McCubrey, Akshaya K. Meher, Shaw M. Akula, Stephen L. Abrams, Linda S. Steelman, Michelle M. LaHair, Richard A. Franklin, Alberto M. Martelli, Stefano Ratti, Lucio Cocco, Fulvio Barbaro, Przemysław Duda, Agnieszka Gizak

**Affiliations:** 1 Department of Microbiology and Immunology, Brody School of Medicine at East Carolina University, Greenville, NC 27858, USA; 2 Department of Biomedical and Neuromotor Sciences, Università di Bologna, Bologna, Italy; 3 Department of Medicine and Surgery, Re.Mo.Bio.S. Laboratory, Anatomy Section, University of Parma, Parma, Italy; 4 Department of Molecular Physiology and Neurobiology, University of Wroclaw, Wroclaw, Poland

**Keywords:** TP53, targeted therapy, PDAC, chemotherapeutic drugs, metabolic properties

## Abstract

TP53 is a master regulator of many signaling and apoptotic pathways involved in: aging, cell cycle progression, gene regulation, growth, apoptosis, cellular senescence, DNA repair, drug resistance, malignant transformation, metastasis, and metabolism. Most pancreatic cancers are classified as pancreatic ductal adenocarcinomas (PDAC). The tumor suppressor gene *TP53* is mutated frequently (50–75%) in PDAC. Different types of TP53 mutations have been observed including gain of function (GOF) point mutations and various deletions of the TP53 gene resulting in lack of the protein expression. Most PDACs have point mutations at the *KRAS* gene which result in constitutive activation of KRas and multiple downstream signaling pathways. It has been difficult to develop specific KRas inhibitors and/or methods that result in recovery of functional TP53 activity. To further elucidate the roles of TP53 in drug-resistance of pancreatic cancer cells, we introduced wild-type (WT) TP53 or a control vector into two different PDAC cell lines. Introduction of WT-TP53 increased the sensitivity of the cells to multiple chemotherapeutic drugs, signal transduction inhibitors, drugs and nutraceuticals and influenced key metabolic properties of the cells. Therefore, TP53 is a key molecule which is critical in drug sensitivity and metabolism of PDAC.

## INTRODUCTION

### Pancreatic cancer—A disease associated with aging that is diagnosed late in development and difficult to successfully treat

When a patient is diagnosed with pancreatic cancer, the outcome is poor [1–7]. There are four stages of pancreatic cancer. This cancer is often detected at stage IV, the most advanced stage [1, 2, 5]. The age of the patient will influence the survival rate as younger pancreatic cancer patients (15–49 years old) have a better survival rate than the older patients (50 and above) [7]. Thus, pancreatic cancer is a disease associated with aging [8, 9].

### Therapeutic approaches for pancreatic cancer

Most pancreatic cancers consist of pancreatic ductal adenocarcinoma (PDAC). They are often refractive to classical chemotherapeutic drugs. PDAC patients undergo surgery to remove the diseased part of the pancreas. However, as PDAC is frequently diagnosed late in the course of the disease, the PDAC has often metastasized to other organs making therapy difficult and ineffective [10–12]. PDACs are often refractive to chemotherapeutic drugs and have modest effects in terms of treatments of the disease [13–17].

### Genes implicated in PDAC

Many genes have been implicated in PDAC including *KRAS*, *TP53*, *CDKN2A*, *SMAD4* and *PDGFβR* [3, 8, 9, 18–22]. Changes in the expression of these genes has many different effects and contribute to PDAC progression and metastasis [23–26]. The *TP53* gene can become mutated by various genetic mechanisms. Two of the most common mechanisms of mutation of *TP53* are point mutations and deletions of part or the entire TP53 gene. Certain point mutations at the *TP53* gene alter the activity of the TP53 protein and give the TP53 protein different properties. This class of *TP53* mutation is referred to as a gain-of-function (GOF) mutation [27–31]. Another class of *TP53* mutation results in the lack of expression of the TP53. This class of *TP53* mutation is referred to TP53-null [27–31].

**Figure 1:**
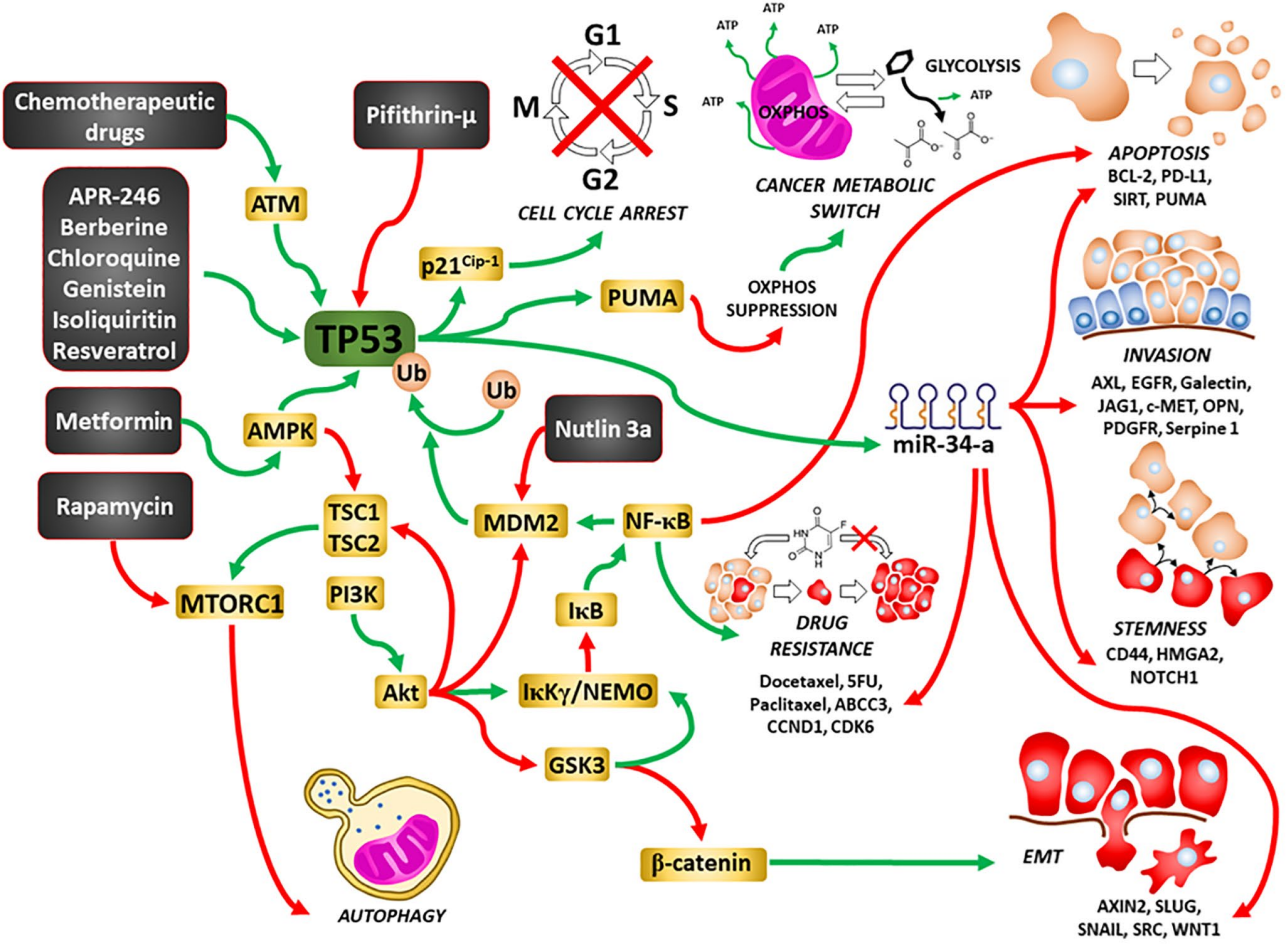
**Illustration of TP53’s interactions with other signaling pathways important in regulation of cell growth and sites of interaction for chemotherapeutic drugs, certain signal transduction inhibitors, natural products and nutraceuticals.** Green arrows = induction of a pathway, red arrows = suppression of a pathway.

Interestingly, a novel class of compounds have been developed which alter the structure of mutant TP53 and restore some of its properties which are important is suppression of cell growth [32, 33]. APR-246 is one such compound which has been examined in clinical trials. A summary of the effects of TP53 on various processes important in cell growth and metastasis is present in Figure 1.

*KRAS* is another important gene which in mutated in >90% of PDAC. Although many potential Ras inhibitors were developed over the past 25 years, they were not specific to mutant KRas, recently, some have shown promise [34, 35]. As with most drugs, cancer cells have developed mechanisms to become resistant to these inhibitors [36]. In the following studies, we examined the consequences of introduction of WT-TP53 gene in two PDAC cell lines, one lacking TP53 expression (TP53-null) and one cell line with GOF-TP53 [37–39]. Earlier studies performed by us, indicated that inheritance of WT-TP53 increased the ability of some chemotherapeutic drugs, signal transduction inhibitors and natural products to inhibit cell proliferation [40, 41]. In the current studies, we examined the effects of inheritance of WT-TP53 on a larger panel of chemotherapeutic drugs as well the consequences of on other properties important in cancer progression such as clonogenicity, colony formation in soft agar and metabolic properties.

## RESULTS

### Restoration of WT-TP53 activity results in decreased resistance to various drugs, inhibitors, and natural products

MIA-PaCa-2 cells have GOF mutant TP53 alleles (R248W). A cDNA encoding WT-TP53 cDNA was inserted into the pLXSN vector [42]. MIA-PaCa-2 cells were transduced with the WT-TP53 vector and named MIA-PaCa-2 + WT-TP53 cells. As a negative control, the effects of the empty parental pLXSN plasmid [43] on MIA-PaCa-2 cells and named MIA-PaCa-2 + pLXSN.

Table 1 is a list of the various agents examined in this study as well as their targets and intersections with the TP53 pathway and a brief description of their mechanisms of action.

**Table 1:** Chemotherapeutic drugs, signal transduction inhibitors, natural products used in this study and their targets, mode of action, and intersections with the TP53 pathway.^1,^^2^

**Chemotherapeutic drugs^1^**			
**Drug↓**	**Target^1^**	**Mode of action**	**Intersection with TP53 pathway**
Docetaxel	Microtubule Binder	Blocks mitosis by inhibiting mitotic spindle assembly.	Docetaxel intersects with TP53 pathway. WT-TP53 increases sensitivity, increases phosphorylation of S15-TP53.
5-Fluorouracil (5FU)	Nucleoside Analogue	Blocks the activity of thymidylate synthase, thus, inhibits DNA synthesis/replication.	5FU intersects with TP53 pathway. WT-TP53 increases sensitivity to FU. 5FU induces TP53 stabilization by blocking MDM2.
Gemcitabine (Gem)	Nucleoside Analogue	Gemcitabine exerts it antitumor effects by promoting apoptosis of cells undergoing DNA synthesis.	Gem intersects with TP53 pathway. WT-TP53 increases sensitivity. Gem can induce TP53 targets such as PUMA and Bax which leads to apoptosis.
Aclacinomycin (Aclarubicin)	DNA intercalator, Topoisomerase II	Topoisomerase inhibitor (inh.) thus, inhibits DNA replication.	As an anthracycline it probably insects with TP53 pathway. However, like most chemotherapeutic drugs, it can function in TP53 mutant cells.
Daunorubicin	DNA intercalators, Topoisomerase II	Topoisomerase inh. thus, inhibits DNA replication.	Daunorubicin intersects with TP53 pathway. It induces TP53 and downstream p21Cip1.
Doxorubicin (Dox)	DNA intercalator, Topoisomerase II	Topoisomerase inh. thus, inhibits DNA replication and induces many TP53-regulated genes, many induce apoptosis.	Dox intersects with TP53 pathway. It increases TP53 expression and phosphorylation at S15 and can induce p21Cip-1.
Etoposide	Binds to Topoisomerase II	Topoisomerase inh. thus, inhibits DNA replication and induces apoptosis. Complex form between etoposide and DNA and can prevent DNA repair.	Etoposide intersects with TP53 pathway. It increases TP53 and pro-apoptotic PUMA expression as well as Bax processing.
Cisplatin (Cis)	DNA	Crosslinks DNA to form DNA adducts. Preventing repair of DNA leading to DNA damage and subsequently apoptosis.	Cis intersects with TP53 pathway. Cis can enhance TP53, p21Cip-1, MDM2 and Fas expression.
**Signal transduction inhibitors^1^**			
**Drug↓**	**Target**	**Mode of action**	**Intersection with TP53 pathway**
ARS-1620	Mutant KRas	KRas-mediated catalysis of the chemical reaction with Cys12 in KRASG12C.	KRas interacts with the TP53 pathway. TP53 and KRas interact to modulate CREB1 expression to promote metastasis and tumor growth.
PD0325901	MEK1	A highly selective allosteric inh. that does not compete with either ATP or ERK1/2.	MEK1 interacts with the TP53 pathway. Downstream ERK can phosphorylate and activate TP53, resulting in many cellular responses.
LY294002	PI3K and others	Competition with ATP for binding the PI3K active site.	PI3K and downstream molecules can interact with the TP53 pathway. Downstream of PI3K are PTEN and Akt and they can regulate the TP53 pathway at various steps and processes.
Pifithrin-μ	TP53	Inhibits some of TP53 activities by binding to BCLXL and BCL2 at the mitochondria without affecting TP53 transcriptional activities.	Pifithrin-μ inhibits some proteins regulated by the TP53 pathway (BCL-XL and BCL2).
6-bromoindirubin-30-oxime (BIO)	GSK-3	BIO is a selective, reversible potent GSK-3 inh. It is an ATP-competitive inhibitor of GSK-3α/β. It interacts with ATP binding site of GSK-3.	GSK-3 interacts with TP53 pathway. GSK-3 phosphorylates sites on the proteasomal inhibitor MDM2. This phosphorylation is required for TP53 degradation. Inhibition of GSK-3 leads to an increase in TP53 levels.
SB415286	GSK-3	Targets ATP-binding site. It inhibits both GSK-3α and GSK-β.	GSK-3 interacts with TP53 pathway. GSK-3 phosphorylates sites on the proteasomal inhibitor MDM2. This phosphorylation is required for TP53 degradation. Inhibition of GSK-3 leads to an increase in TP53 levels.
CHIR99021	GSK-3	Targets ATP-binding site. It inhibits both GSK-3α and GSK-β.	GSK-3 interacts with TP53 pathway. GSK-3 phosphorylates sites on the proteasomal inhibitor MDM2. This phosphorylation is required for TP53 degradation. Inhibition of GSK-3 leads to an increase in TP53 levels.
Rapamycin	mTORC1	Binds and blocks mTORC1 complex.	mTORC1 interacts with the TP53 pathway. Activation of TP53 downregulates mTOR signaling. This occurs through AMPK.
AG1498	EGFR	AG1478 competitively binds to the ATP binding pocket in EGFR.	EGFR interacts with the TP53 pathway.TP53 mutations are associated with primary or acquired resistance to EGFR-tyrosine kinase inhibitors.
Gilteritinib	AXL/ALK/FLT3	Gilteritinib binds to the ATP binding site in the active pocket of the AXL/ALK/FLT3 kinases.	AXL/ALK/FLT3 interacts with the TP53 pathway. AXL suppresses TP53 expression by binding to DNA sequences upstream from the TP53 gene. AXL is also regulated by miR-34a which is regulated by TP53. ALK inhibitors are not as effective in lung cancer patients that have rearranged ALK genes and are also mutated at TP53 as in patients with germline genes. Also, FLT-3 and TP53 also interact.
Sorafenib	Multiple kinases (e.g., Raf, PDGFR, VEGFR, FLT-3 and others)	Sorafenib binds to the ATP binding site.	Many of these kinases and their downstream substrates interact with TP53 pathway by phosphorylating TP53 and other molecules regulated by TP53. Mutant TP53 can also regulate the expression of some of these kinases such as PDGFR.
OTX008	Galectin-1	OTX008 binds galectin-1 which leads to galectin-1 oxidation and proteasomal degradation.	Galectin-1 can interact with the TP53 pathway. TP53 can induce the expression of miRs which regulate galectin-1 expression.
Tiplaxtinin	Serpine-1	Tiplaxtinin binds to the active conformation of serpine-1 and induced reversible inactivation serpine-1.	TP53 regulates the expression of miR-34a which can down regulate serpine-1. Serpine1- is involvement of metastasis in various cancers.
Verapamil (Ver)	Calcium channel	Also, some transporters associated with chemotherapeutic drug resistance. Binds to sites on MDR1 glycoprotein preventing drug efflux. Also, downregulates MDR1 expression.	TP53 pathway and Ver interact. Ver interacts with the TP53 activator (MDM2 inhibitor) nutlin-3a which results in suppression of cell growth.
Vismodegib (Vis)	Hh pathway	Smoothened homologue (SMO) binds to Smoothened (SMO) and inhibits its activity.	Multiple interactions with TP53 pathway.
**Natural products^2^**			
Cyclopamine	Sonic hedgehog (SHH) pathway	Cyclopamine binds to SMO and inhibits its activity.	Multiple interactions with TP53 pathway.
Parthenolide^2^	NF-κB (other targets)	Inhibition of activation of IκB, and direct binding to NF-κB, preventing its interaction with DNA.	NF-κB interacts with the TP53 pathway TP53 and NF-κB inhibit each other’s ability to stimulate gene expression.
Isoliquiritin^2^	Induces apoptotic cell death through upregulating TP53 and p21Cip-1. Suppresses NF-κB, ERK and activation of other targets	Suppresses invasiveness and angiogenesis of cancer.	Isoliquiritin interacts with TP53 pathway. It induces TP53 and inhibits NF-κB and ERK. Both interact with TP53 pathway.
Genistein (isoflavone)^2^	Multiple targets	Genistein triggers the ER stress to induce apoptosis and other mechanisms of cell death.	Genistein interacts with TP53 pathway. Genistein increases the phosphorylation and activation of ATM/ATR-TP53-p21Cip-1 pathway.
Daidzein (isoflavone)^2^	Multiple targets	Daidzein and genistein induce cell cycle arrest in the G2/M phase. This is accompanied by activation of ATM/TP53, and p21Cip-1 and other cell cycle regulatory genes.	Daidzein interacts with TP53 pathway. Daidzein increases the phosphorylation and activation of ATM/ATR-TP53-p21Cip-1 pathway.

^1^Many chemotherapeutic drugs and signal transduction inhibitors have other effects and targets. We describe the targets that are most closely related to TP53.

^2^Most natural products have multiple targets. We describe some of the targets that are more closely related to TP53.

**Figure 2:**
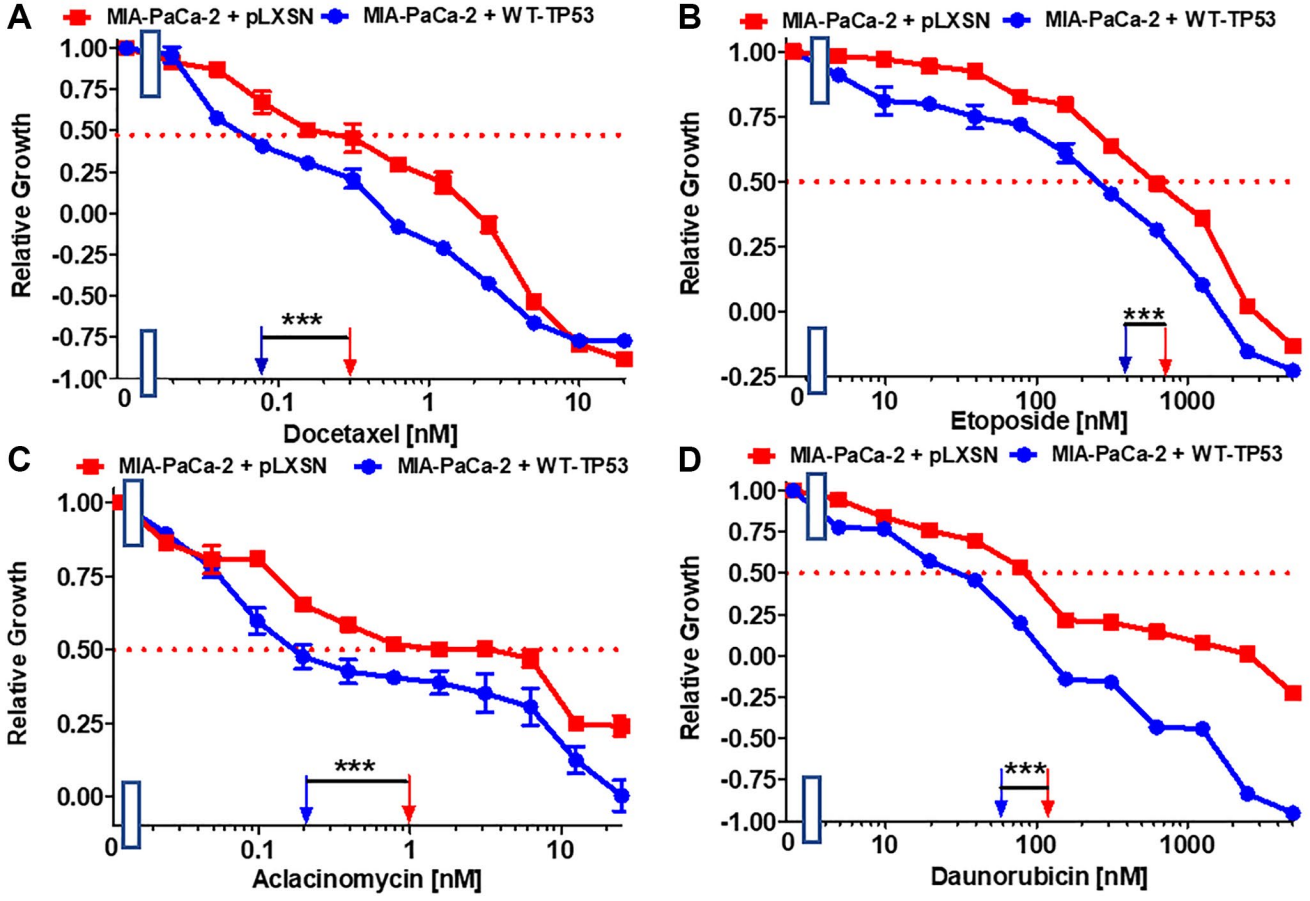
**Effects of signal transduction inhibitors on the growth of MIA-PaCa-2 + WT-TP53 and MIA-PaCa-2 + pLXSN cells.** The effects of docetaxel (**A**), etoposide (**B**) aclacinomycin (**C**) and daunorubicin (**D**) on MIA-PaCa-2 + pLXSN cells (solid red squares) and MIA-PaCa-2 + WT-TP53 cells (solid blue circles) were examined by MTT analysis. These experiments were repeated and similar results were obtained. Statistical analyses were performed by the Student *T* test on the means and standard deviations of various treatment groups. ^***^P < 0.0001.

Docetaxel is a common chemotherapeutic drug used to treat various cancer types including PDAC. The IC_50_ for docetaxel in MIA-PaCa-2 + WT-TP53 cells was 3.8-fold lower than in MIA-PaCa-2 + pLXSN cells (Figure 2A). The effects of WT-TP53 on the sensitivity to three topoisomerase inhibitors used in cancer therapy were also examined (Figure 2B–2D). The IC_50_s for all the inhibitors were lower (~ 2-fold for etoposide and daunorubicin, and 5-fold for aclacinomycin) in MIA-PaCa-2 + WT-TP53 cells than in MIA-PaCa-2 + pLXSN cells.

**Figure 3:**
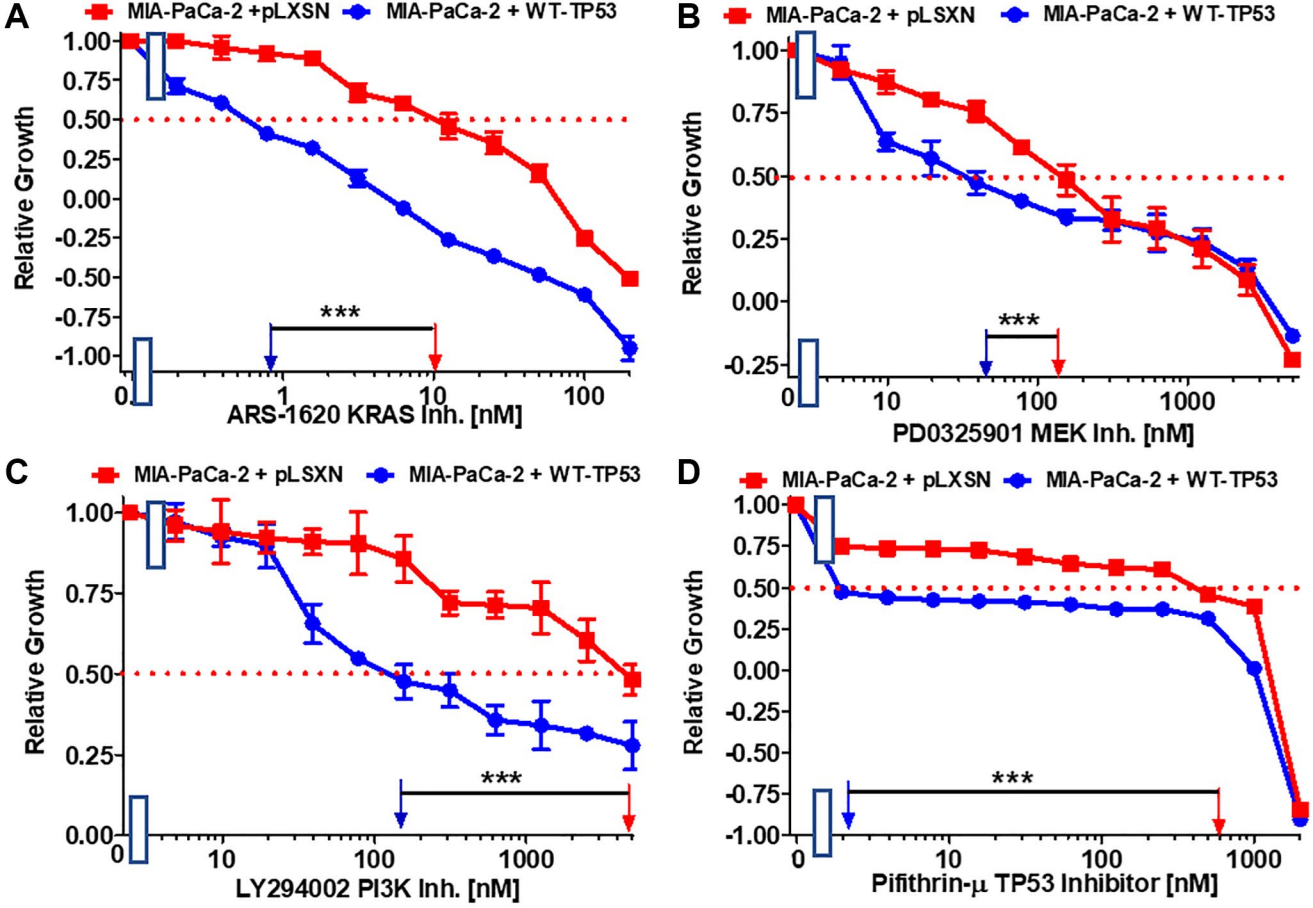
**Effects of the Ras/MEK, PI3K/mTOR and TP53 inhibitors on the growth of MIA-PaCa-2 + WT-TP53 and MIA-PaCa-2 + pLXSN cells.** The effects of the ARS-1620 mutant KRas inhibitor (**A**), the PD0325901 MEK1 inhibitor (**B**), the LY294002 PI3K inhibitor (**C**) and the TP53 inhibitor pifithrin-μ (**D**) on MIA-PaCa-2 + pLXSN cells (solid red squared) and MIA-PaCa-2 + WT-TP53 cells (solid blue circles) were examined by MTT analysis. The MIA-PaCa-2 + WT-TP53, and MIA-PaCa-2 + pLXSN cells in each panel were all examined at the same time period. These experiments were repeated and similar results were obtained. Statistical analyses were performed by the Student *T* test on the means and standard deviations of various treatment groups. ^***^P < 0.0001.

Restoration of WT-TP53 activity in MIA-PaCa-2 cells resulted in increased sensitivity to chemotherapeutic drugs used to treat cancer patients. Table 2 summarizes the effects of addition of WT-TP53 into Mia-PaCa-2 cells. Restoration of WT-TP53 activity increased sensitivity to the KRAS inhibitor ARS-1620 [44] 125-fold (Figure 3A).

**Table 2:** Effects of WT-TP53 and pLXSN on sensitivity of MIA-PaCa-2 pancreatic cancer cells on chemotherapeutic drugs, signal transduction inhibitors and natural products as determined by IC_50_ analysis.^1^

**Drug/Agent↓**	**+ pLXSN**	**+ WT-TP53**	**Fold change WT vs. LXSN**
Docetaxel (microtubule binder)	0.3 nM	0.08 nM	3.8 X↓
Etoposide (topoisomerase inh.)	750 nM	400 nM	1.9 X↓
Aclacinomycin (topoisomerase inh.)	1 nM	0.2 nM	5 X↓
Daunorubicin (topoisomerase inh.)	120 nM	60 nM	2 X ↓
ARS-1620 (mutant KRas inh.)	10 nM	0.8 nM	12.5 X↓
PD0325901 (MEK1 inh.)	150 nM	45 nM	3.3 X↓
LY294002 (PI3K inh.)	5,000 nM	150 nM	33.3 X↓
Pifithrin-μ (TP53 inh.)	600 nM	2.5 nM	240 X↓
BIO (GSK-3 inh.)	210 nM	100 nM	2.1 X↓
SB415286 (GSK-3 inh.)	40 nM	3 nM	13.3 X↓
CHIR99021 (GSK-3 inh.)	500 nM	300 nM	1.7 X↓
Rapamycin (mTORC1 blocker)	2 nM	0.3 nM	6.7 X↓
AG1498 (EGFR inh.)	1,000 nM	200 nM	5 X↓
Gilteritinib (AXL/ALK/FLT3 inh.)	600 nM	220 nM	2.7 X↓
Sorafenib (multi-kinase inh.)	1,000 nM	700 nM	1.4 X↓
OTX008 (Galectin-1 inh.)	1,000 nM	10 nM	100 X↓
Tiplaxtinin (Serpine-1 inh.)	40 nM	10 nM	4 X↓
Cyclopamine (SHH inh.)	1,000 nM	500 nM	2 X↓
Parthenolide (NF-κB inh, other targets)	40 nM	3.5 nM	11.4 X↓
Isoliquiritin (multiple targets)	1,900 nM	600 nM	3.2 X↓
Genistein (isoflavone, many targets)	300 nM	70 nM	4.3 X↓
Daidzein (isoflavone, many targets)	1,000 nM	600 nM	1.7 X↓

^1^Determined by MTT analysis as previously described [40, 41].

Various signaling cascades are located downstream of KRas. Two important kinase cascades are the Raf/MEK/ERK and PI3K/PTEN/Akt/mTORC1 pathways. They are often involved in regulation of cell growth and their aberrant regulation is often implicated in cancer [45–47]. Restoration of WT-TP53 activity in MIA-PaCa-2 cells increased the sensitivity to the MEK1 inhibitor PD0325901 3.3-fold. (Figure 3B).

Restoration of WT-TP53 activity in MIA-PaCa-2 cells led to a 33.3-fold lower IC_50_ for the PI3K inhibitor LY294002 inhibitor than that observed in MIA-PaCa-2 cells lacking WT-TP53 (Figure 3C). Thus, addition of WT-TP53 activity in MIA-PaCa-2 cells increased their sensitivity to small molecule inhibitors which target the Ras/Raf/MEK/ERK and PI3K/PTEN/Akt/mTORC1 pathways.

Pifithrin-μ is a small molecule that inhibits the interactions of TP53 with either BCL2 or BCLXL at the mitochondrial membrane. This results in the induction of apoptosis. However, Pifithrin-μ does not inhibit the effects that TP53 has on transcription [48]. Restoration of WT-TP53 in MIA-PaCa-2 cells resulted in a pifithrin-μ IC_50_ 240-fold lower than that detected in MIA-PaCa-2 cells which lack WT-TP53 activity (Figure 3D).

**Figure 4:**
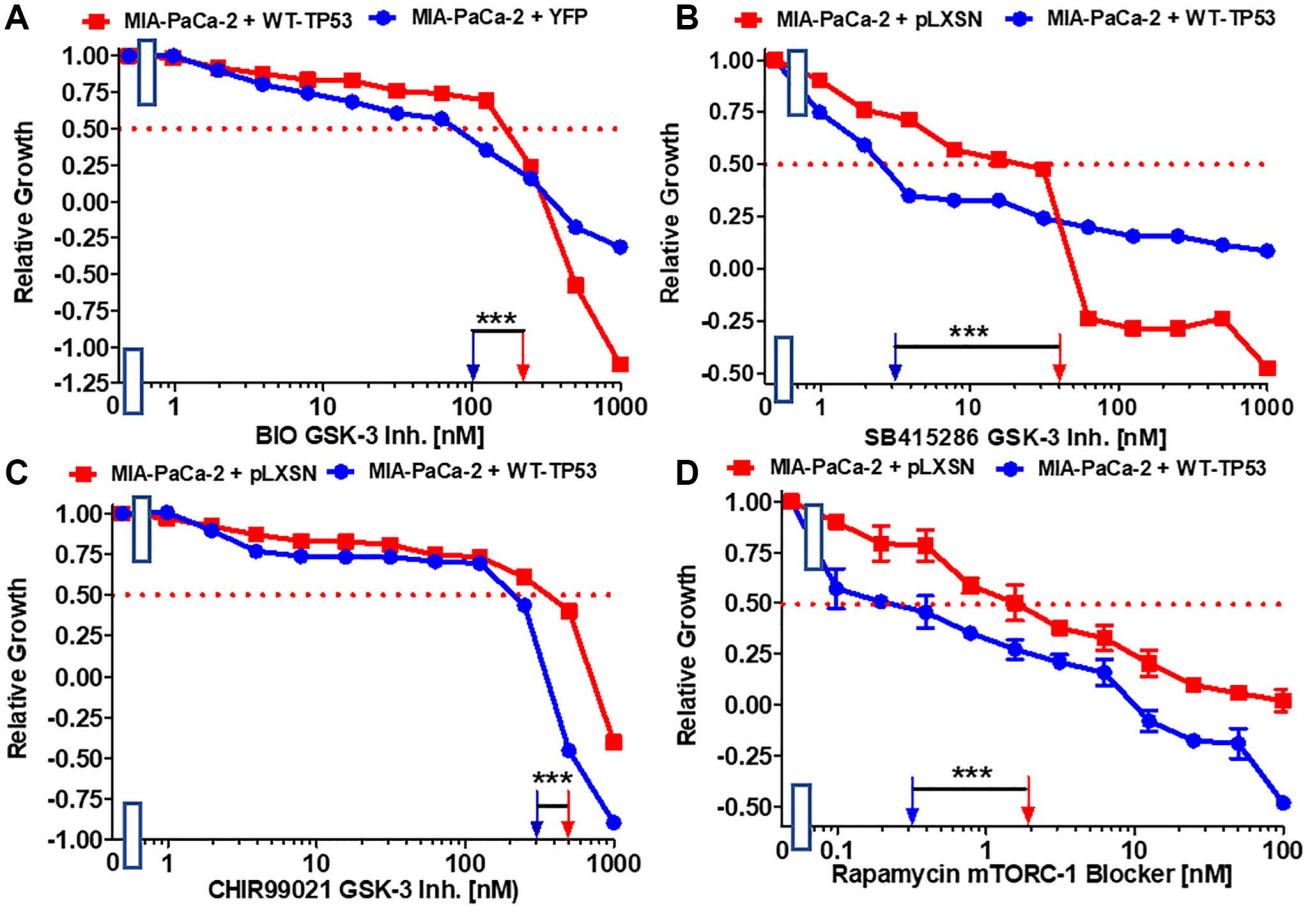
**Effects of GSK-3 inhibitors and the mTORC1 blocker rapamycin on the growth of MIA-PaCa-2 + WT-TP53 and MIA-PaCa-2 + pLXSN cells.** The effects of the BIO GSK-3 inhibitor (**A**), the SB415286 GSK-3 inhibitor (**B**), the CHIR99021 GSK-3 inhibitor (**C**) and the mTORC1 blocker rapamycin (**D**) on MIA-PaCa-2 + pLXSN cells (solid red squared) and MIA-PaCa-2 + WT-TP53 cells (solid blue circles) were examined by MTT analysis. The MIA-PaCa-2 + WT-TP53, and MIA-PaCa-2 + pLXSN cells in each panel were all examined at the same time period. These experiments were repeated and similar results were obtained. Statistical analyses were performed by the *T* test on the means and standard deviations of various treatment groups. ^***^P < 0.0001.

GSK-3 is a multifunctional kinase that is involved in the regulation of many processes both in normal physiological and malignant growth [49]. GSK-3 has been shown to be important for the interactions between KRas and NF-κB [50, 51]. GSK-3 is an important target in many cancers. GSK-3 inhibitors have been suggested for the treatment of PDAC [52]. The effects of GSK-3 inhibitors BIO, CHIR99021 and SB415286 on MIA-PaCa-2 cells containing and lacking WT activity were examined. Restoration of WT-TP53 activity in MIA-PaCa-2 cells resulted in over 13-fold lower IC_50_ for SB415286 and only about 2-fold lower IC50 for BIO and CHIR99021 than in cells lacking WT-TP53 activity (Figure 4A–4C).

The mTORC1 complex plays critical roles in many processes, including: cell growth, metabolism, cancer and aging [53, 54]. Restoration of WT-TP53 activity in MIA-PaCa-2 cells resulted in a rapamycin IC_50_ 6.7-fold lower than that observed in cells lacking WT-TP53 activity (Figure 4D).

**Figure 5:**
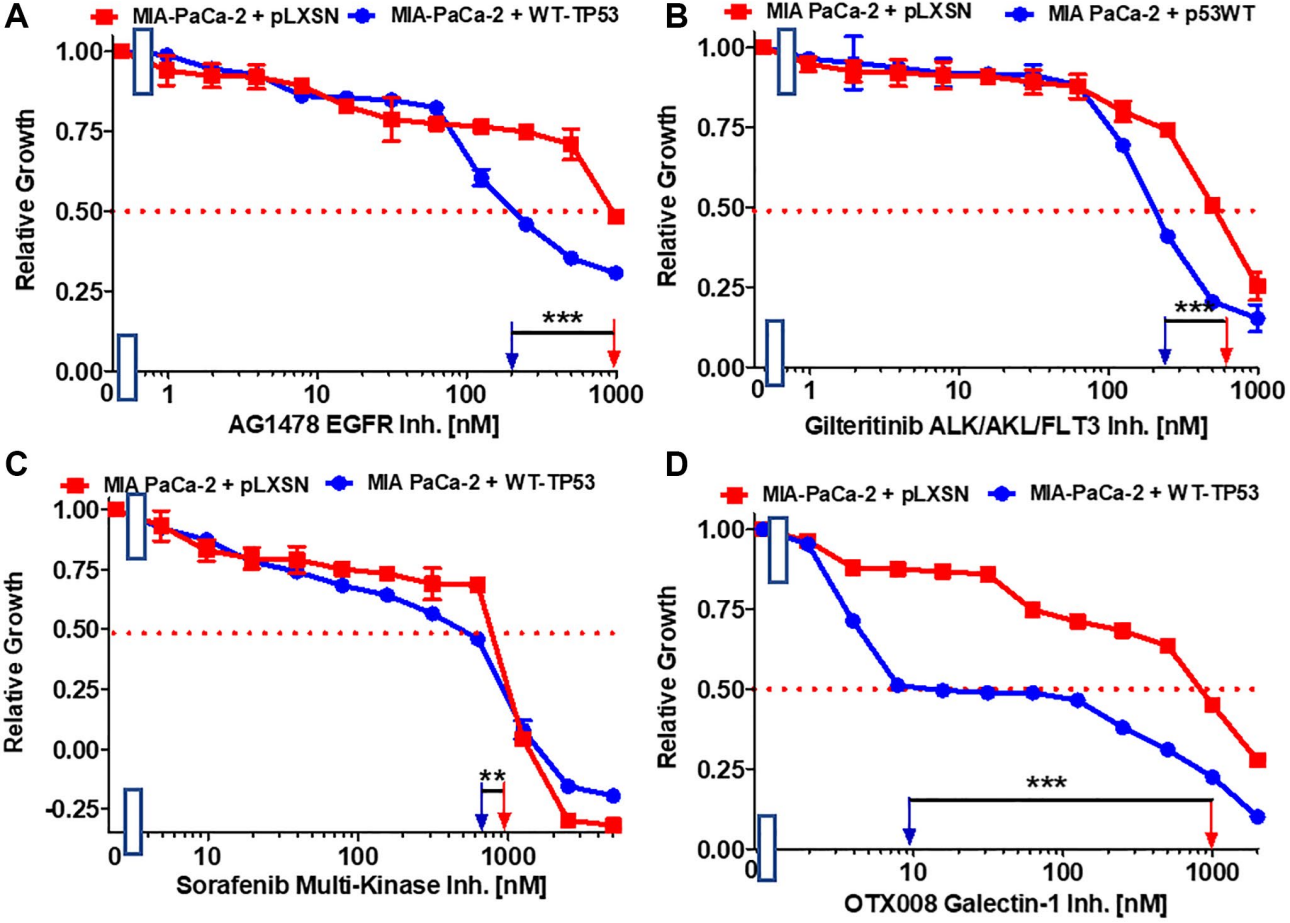
**Effects of inhibitors which may suppress metastasis on the growth of MIA-PaCa-2 + WT-TP53 and MIA-PaCa-2 + pLXSN cells.** The effects of the AG1478 EGFR inhibitor (**A**), the gilteritinib ALK/AXL/FLT3 inhibitor (**B**), the sorafenib multi-kinase inhibitor (**C**) and the galectin-1 inhibitor OTX008 (**D**) on MIA-PaCa-2 + pLXSN cells (solid red squares) and MIA-PaCa-2 + WT-TP53 cells (solid blue circles) were examined by MTT analysis. The MIA-PaCa-2 + WT-TP53, and MIA-PaCa-2 + pLXSN cells in each panel were all examined at the same time period. These experiments were repeated and similar results were obtained. Statistical analyses were performed by the *T* test on the means and standard deviations of various treatment groups. ^***^P < 0.0001, and ^**^P < 0.005.

EGFR, HER2, ALK, AXL, FLT3, PDGFR and other receptors and signal transducers (*e.g*., Raf) are involved in the metastasis of various cancers [55–61]. The effects of: the AG1478 EGFR inhibitor, the multi-kinase ALK, AXL, FLT3 inhibitor gilteritinib and multi-kinase Raf, PDGFR, FLT3, VEGFR inhibitor sorafenib on the growth of MIA-PaCa-2 cells expressing WT-TP53 or not were ascertained. Introduction of WT-TP53 into MIA-PaCa-2 cells resulted in reduction of the IC_50_s for all the inhibitors in comparison to the IC_50_s in MIA-PaCa-2 cells lacking WT-TP53 expression (Figure 5A–5C) but the reduction was most pronounced for the AG1478 EGFR inhibitor.

Galectin-1 is involved in hedgehog (Hh) signaling, stromal remodeling and metastasis of PDAC [62]. Galectin-1 is negatively regulated by WT TP53 [63]. OTX008 inhibits the activity of galectin-1. Restoration of WT-TP53 activity in MIA-PaCa-2 cells sensitized the cells 100-fold in comparison to MIA-PaCa-2 cells which lacked WT-TP53 activity (Figure 5D).

**Figure 6:**
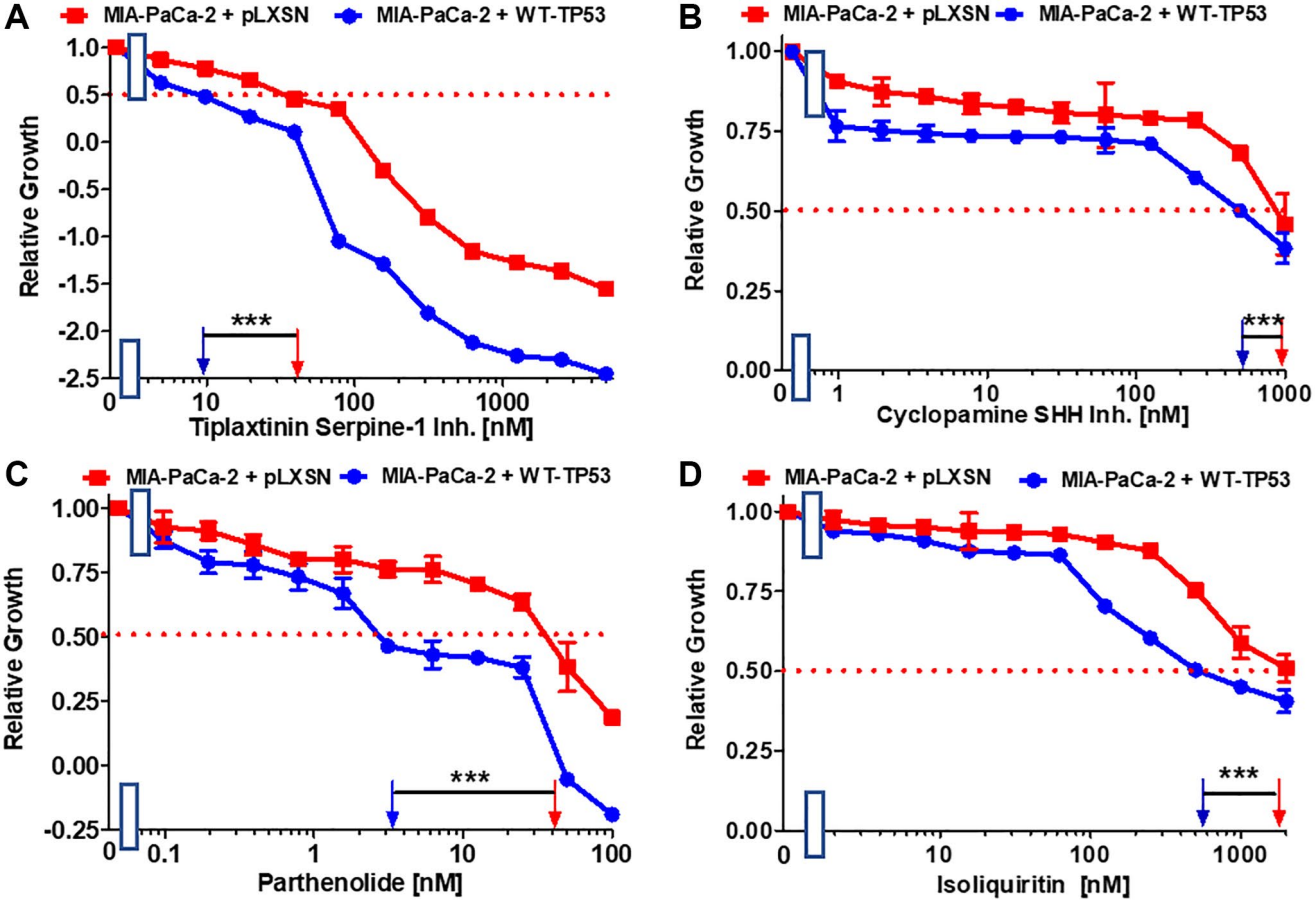
**Effects of inhibitors/natural products which may suppress metastasis on the growth of MIA-PaCa-2 + WT-TP53 and MIA-PaCa-2 + pLXSN cells.** The effects of the tiplaxtinin Serpine-1 inhibitor (**A**), the natural product cyclopamine, a SHH inhibitor (**B**), the natural product parthenolide, a NF-κB inhibitor (**C**), and the natural product/nutraceutical isoliquiritin (**D**) were examined by MTT analysis. The MIA-PaCa-2 + WT-TP53, and MIA-PaCa-2 + pLXSN cells in each panel were all examined at the same time period. These experiments were repeated and similar results were obtained. Statistical analyses were performed by the *T* test on the means and standard deviations of various treatment groups. ^***^P < 0.0001.

The plasminogen activator inhibitor (PAI-1), serpine1 is negatively regulated by miR-34a in MIA-PaCa-2 upon restoration of WT-TP53 activity [64]. The small molecule tiplaxtinin inhibits serpine1 activity [65]. Upon restoration of WT-TP53 activity in MIA-PaCa-2 cells resulted in 4-fold enhanced sensitivity to tiplaxtinin in comparison to MIA-PaCa-2 cells lacking WT-TP53 activity (Figure 6A).

### Effects of WT-TP53 on sensitivity to natural products and nutraceuticals

The ability of various natural products and nutraceuticals to inhibit the proliferation in MIA-PaCa-2 cells in the presence and absence of WT-TP53 activity was determined. These compounds were selected on the basis of literature data suggesting their targets and their influence on the development of PDAC.

A natural product that inhibits the Hh signaling pathway is cyclopamine. The Hh pathway is very important in PDAC and metastasis [66, 67]. Restoration of WT-TP53 activity in MIA-PaCa-2 cells increased the sensitivity 2-fold to cyclopamine (Figure 6B).

Extracts from the plant fever few contain parthenolide. One of its targets is NF-κB [68]. Parthenolide has been observed to suppress PDAC progression [69]. Restoration of WT-TP53 activity in MIA-PaCa-2 cells increased the sensitivity to parthenolide 11.4-fold in comparison to cells lacking WT-TP53 activity (Figure 6C).

Licorice contains the flavonoid isoliquiritin which has various biological activities including anti-cancer activities [70, 71]. In lung cancer cells, it was shown that isoliquiritin can induce TP53 activity [71]. In pancreatic cancer cells it suppressed the invasiveness *in vitro* [72]. Restoration of WT-TP53 activity in MIA-PaCa-2 cells increased their sensitivity to isoliquiritin 3.2-fold (Figure 6D).

**Figure 7:**
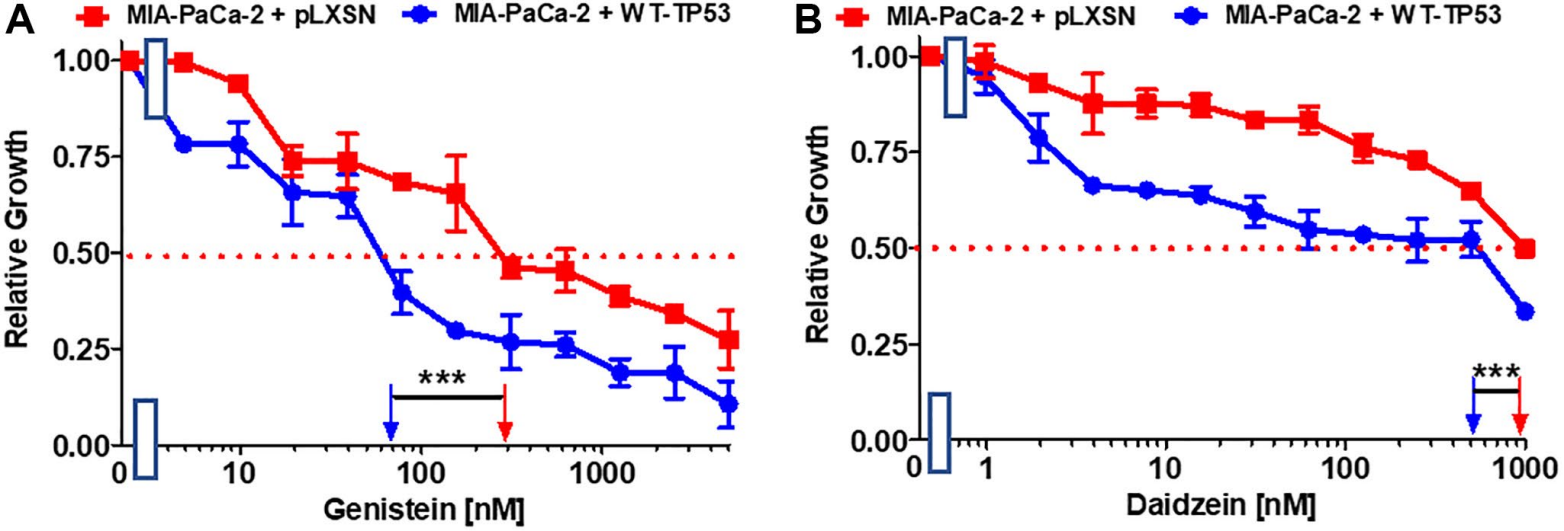
**Effects of nutraceuticals on the growth of MIA-PaCa-2 + WT-TP53 and MIA-PaCa-2 + pLXSN cells.** The effects of genistein (**A**), and daidzein (**B**), on MIA-PaCa-2 + pLXSN cells (solid red squared) and MIA-PaCa-2 + WT-TP53 cells (solid blue circles) were examined by MTT analysis. The MIA-PaCa-2 + WT-TP53, and MIA-PaCa-2 + pLXSN cells in each panel were all examined at the same time period. These experiments were repeated and similar results were obtained. Statistical analyses were performed by the *T* test on the means and standard deviations of various treatment groups. ^***^P < 0.0001.

Genistein is an isoflavone. It possesses certain anti-cancer properties including inhibition of angiogenesis in PDAC [73]. It induces apoptosis in PDAC lines [74]. Restoration of WT-TP53 activity in MIA-PaCa-2 cells increased their sensitivity to genistein 4.3-fold in comparison to MIA-PaCa-2 cells lacking WT-TP53 (Figure 7A).

Daidzein is an additional isoflavone. It inhibited breast cancer growth in rodent models [75, 76]. Restoration of WT-TP53 activity in MIA-PaCa-2 cells increased the sensitivity to daidzein 1.7-fold (Figure 7B).

Summarizing, restoration of WT-TP53 activity in MIA-PaCa-2 cells increased the sensitivity to various chemotherapeutic drugs, signal transduction inhibitors and natural products.

### Restoration of WT-TP53 decreases clonogenicity in the presence of chemotherapeutic drugs

**Figure 8:**
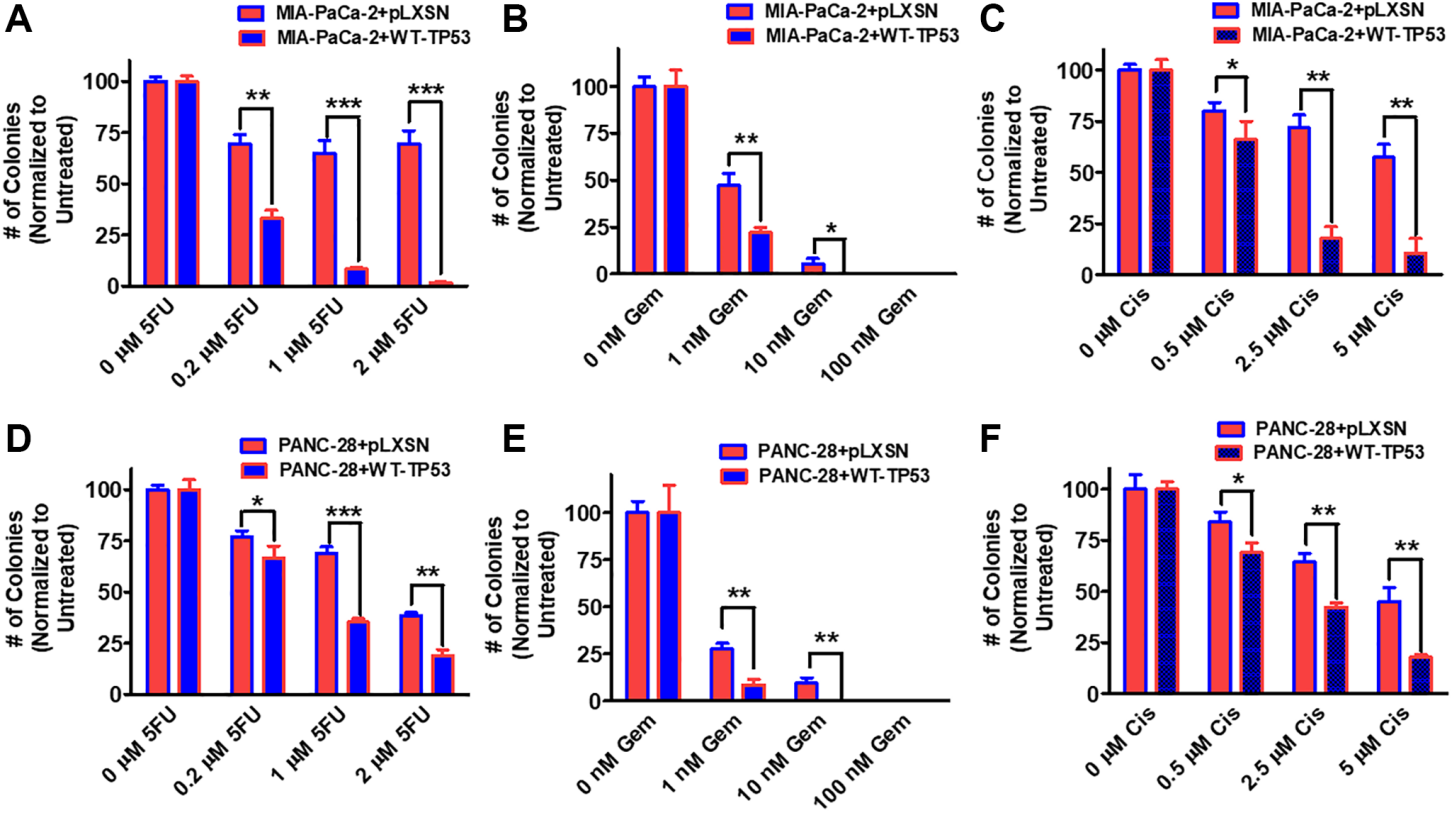
**Effects of pLXSN and WT-TP53 on clonogenicity in the presence of 5-Fluorouracil, gemcitabine or cisplatin in two PDAC cell lines.** The clonogenicity in the presence of increasing concentrations of 5-fluorouracil (5FU), gemcitabine (Gem) and cisplatin (Cis) were examined in: MIA-PaCa-2 + pLXSN and MIA-PaCa-2 + WT-TP53 (**A**–**C**), PANC-28 + pLXSN, and PANC-28 + WT-TP53 (**D**–**F**). Red horizontal bars = MIA-PaCa-2 or PANC-28 containing pLXSN. Blue horizontal bars = MIA-PaCa-2 or PANC-28 containing WT-TP53. These experiments were repeated and similar results were observed. The colonies for each cell line were normalized to untreated so that the results from pLXSN and WT-TP53 could be compared. ^***^P < 0.0001, ^**^P < 0.005 and ^*^P < 0.05.

The ability of WT-TP53 to suppress clonogenicity in 5-fluorouracil, gemcitabine and cisplatin was determined in MIA-PaCa-2 and PANC-28 cell containing and lacking WT-TP53. Upon restoration of WT-TP53 activity in MIA-PaCa-2 and PANC-28 cells, clonogenicity decreased in a dose-dependent fashion more dramatically in cells containing WT-TP53 activity (Figure 8). Although gemcitabine inhibited clonogenicity in cells containing and lacking WT-TP53 activity. Thus, restoration of WT-TP53 suppressed clonogenicity in larger culture volumes containing chemotherapeutic drugs carried out for 14–21 days and it reduced the IC_50_s for chemotherapeutic drugs in smaller cultures carried out over 5 days [40, 41].

### Effects of WT-TP53 on the ability of cells to form colonies in medium containing soft agar

The ability of cells to form colonies in medium containing soft agar in the absence of adhesion to the bottom of the tissue culture plate (anchorage-independent growth) is often considered as a measure of the extent of transformation of malignant transformation as most “normal” cells do not [77].

**Figure 9:**
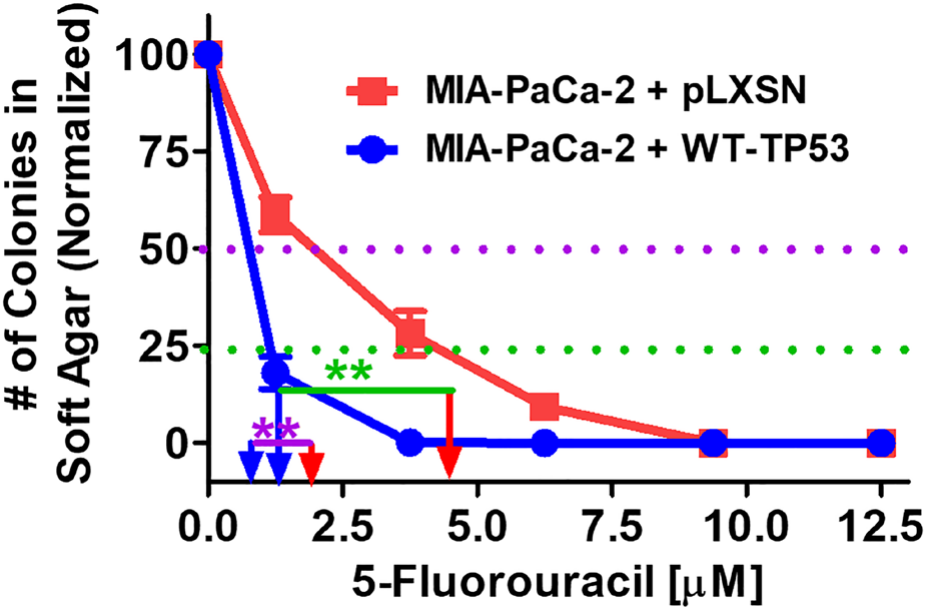
**Effects of pLXSN and WT-TP53 on the colony formation in soft agar in the presence of 5-Fluorouracil.** The effects of pLXSN and WT-TP53 on the colony formation in soft agar were examined. Red squares = MIA-PaCa-2 + pLXSN cells, blue circles = MIA-PaCa-2 + WT-TP53 cells. IC_50_ is indicated with a purple dotted line and IC_25_ is indicated with a green dotted line. IC_25_ is a term to indicate inhibition of colony formation at 25%. These experiments were repeated performed and similar results were observed. The colonies for each cell line were normalized to untreated cells so that the results from the MIA-PaCa-2 + pLXSN and MIA-PaCa-2 + WT-TP53 could be compared. ^**^P < 0.005.

The effects of restoration of WT-TP53 activity on the ability to form colonies in increasing concentrations of 5FU were compared. As documented in Figure 9, restoration of WT-TP53 activity in MIA-PaCa-2 cells inhibited their ability to form colonies in soft agar in the presence of 5FU.

**Figure 10:**
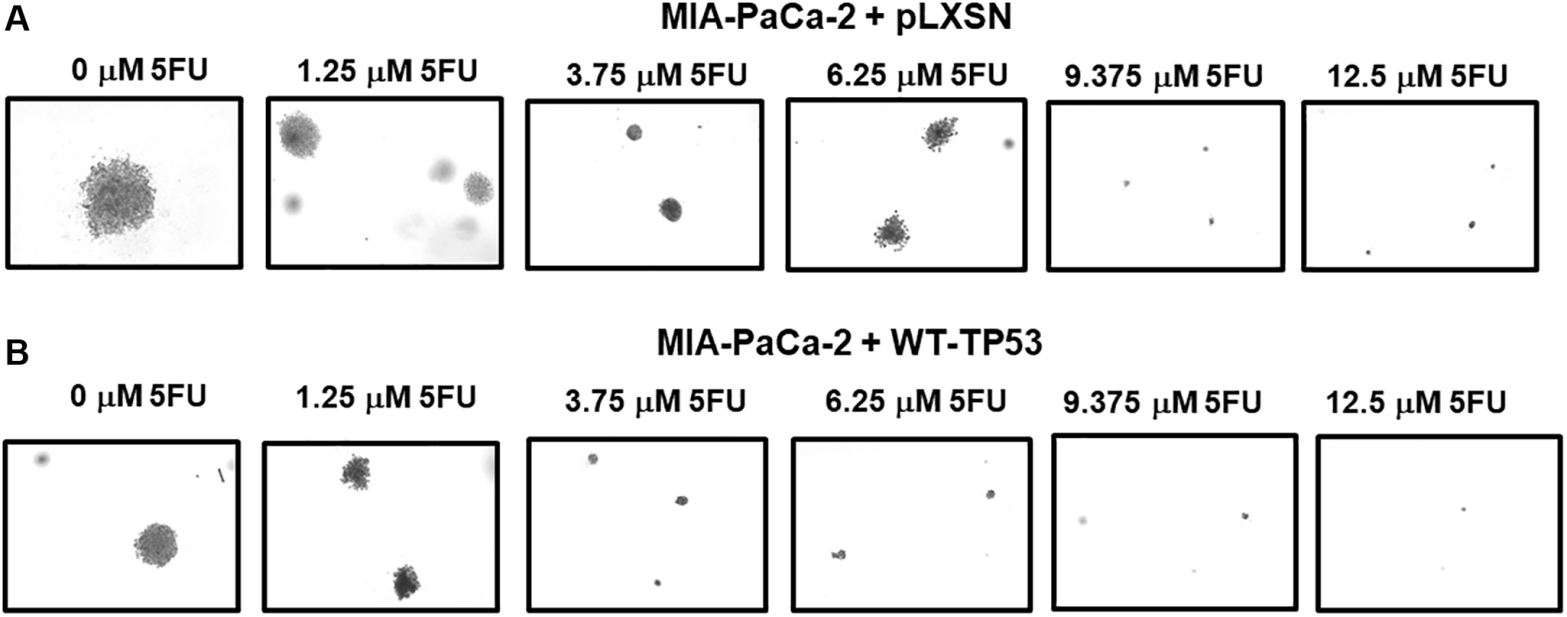
**Crystal violet-stained colonies in soft agar in the presence of 5-Fluorouracil.** The effects of pLXSN and WT-TP53 on the colony formation in soft agar were photographed after staining. Photographs were taken at the same day and at the same magnification on the microscope. (**A**) MIA-PaCa-2 + pLXSN cells treated with increasing concentration of 5FU, (**B**) MIA-PaCa-2 + WT-TP53 cells treated with increasing concentrations of 5FU.

Figure 10 presents photographs of colonies stained with crystal violet, not only were there less colonies in soft agar when WT-TP53 activity was restored to MIA-PaCa-2 cells but the colony sizes were also smaller. When there was no 5FU in the culture medium, MIA-PaCa-2 cells containing or lacking WT-TP53 formed similar numbers of colonies of roughly equal sizes. However, even at the lowest dose of 5FU (1.25 μM), there was a massive drop in the number of colonies observed in MIA-PaCa-2 cells containing WT-TP3 activity while the decline in MIA-PaCa-2 cells lacking WT-TP53 activity, was not as extreme.

**Figure 11:**
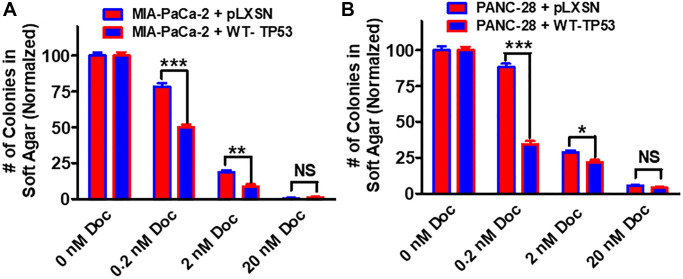
**Effects of pLXSN and WT-TP53 on the colony formation in soft agar in the presence of docetaxel.** The effects of pLXSN and WT-TP53 on the colony formation in soft agar in MIA-PaCa-2 and PANC-28 cells were examined. (**A**) MIA-PaCa-2 + pLXSN (red bars) and MIA-PaCa-2 + WT-TP53 (blue bars) were compared in response to docetaxel. (**B**) PANC-28 + pLXSN (red bars) and PANC-28 + WT-TP53 (blue bars) were compared in response to docetaxel. The colonies for each cell line were normalized to untreated so that the results from pLXSN and WT-TP53 could be compared. These studies were repeated and similar results were observed. ^***^P < 0.0001, ^**^P < 0.005 and ^*^P < 0.05, NS = not statistically significant.

**Figure 12:**
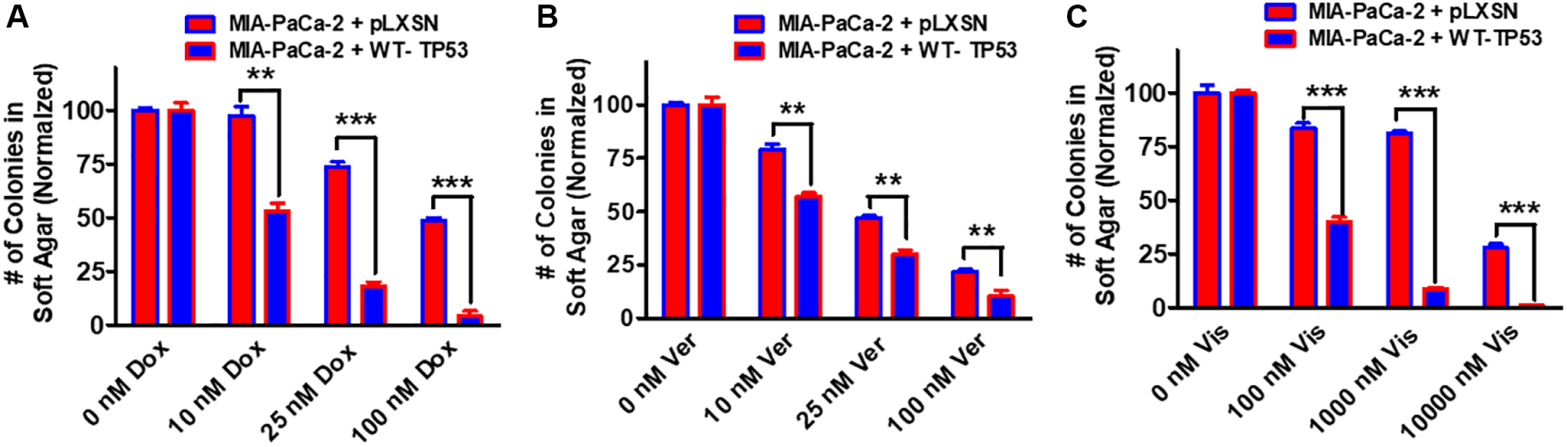
**Effects of pLXSN and WT-TP53 on the colony formation in soft agar in the presence of doxorubicin, verapamil and vismodegib.** The effects of pLXSN and WT-TP53 on the colony formation in soft agar in MIA-PaCa-2 in response to drugs was examined. (**A**) Colony formation abilities of MIA-PaCa-2 + pLXSN (red bars) and MIA-PaCa-2 + WT-TP53 (blue bars) were compared in response to treatment with doxorubicin. (**B**) Colony formation abilities of MIA-PaCa-2 + pLXSN (red bars) and MIA-PaCa-2 + WT-TP53 (blue bars) were compared in response to verapamil. (**C**) Colony formation abilities of MIA-PaCa-2 + pLXSN (red bars) and MIA-PaCa-2 + WT-TP53 (blue bars) were compared in response to treatment with vismodegib. The number of colonies for each cell line were normalized to untreated so that the results from pLXSN and WT-TP53 could be compared. These studies were repeated and similar results were observed. ^***^P < 0.0001, and ^**^P < 0.005.

Restoration of WT-TP53 activity in both MIA-PaCa-2 and PANC-28 cells decreased their ability to form colonies in soft agar containing docetaxel (Figure 11). Introduction of WT-TP53 activity decreased the ability of MIA-PaCa-2 cells to form colonies in soft agar containing doxorubicin (Figure 12A).

Drug transporters such as MDR1 are often upregulated in drug resistant cells [78–80]. Verapamil will inhibit the activity of certain drug transporters such as MDR1. Addition of WT-TP53 activity to MIA-PaCa-2 cells increased their sensitivity to verapamil as determined by colony formation in soft agar (Figure 12B).

Hh signaling is critical in differentiation and in some cases, cancer metastasis [81]. Hh pathway inhibitors have been evaluated in PDAC patients [82]. Restoration of WT-TP53 activity in MIA-PaCa-2 cells made them more sensitive to the Hh pathway vismodegib in soft agar colony formation assays (Figure 12C). Thus, restoration of WT-TP53 activity in both MIA-PaCa-2 and PANC-28 cells resulted in the cells becoming more sensitive to chemotherapeutic drugs.

### Restoration of WT-TP53 activity in MIA-PaCa-2 cells alters their metabolic properties

For their rapid growth, cancer cells require a large amount of ATP that occurs by glycolysis and mitochondrial oxidative phosphorylation. To determine the consequence of restoration of WT-TP53 activity in energy metabolism in MIA-PaCa-2 cells, stress tests were done with the Seahorse analyzer. This machine determines the extent of glycolysis by determining the extracellular acidification (ECAR) and can also analyze mitochondrial oxidative phosphorylation by measuring the real-time oxygen consumption rate (OCR).

TP53 has been shown to be a cellular energy metabolism regulator [83–88]. It can influence both glycolysis and mitochondrial metabolism through multiple mechanisms [88]. Some studies have shown that mutant TP53 can have more effects on mitochondrial metabolism than glycolysis [89]. The effects of restoration of WT-TP53 activity on mitochondrial activity in PDAC cells have not been documented well.

**Figure 13:**
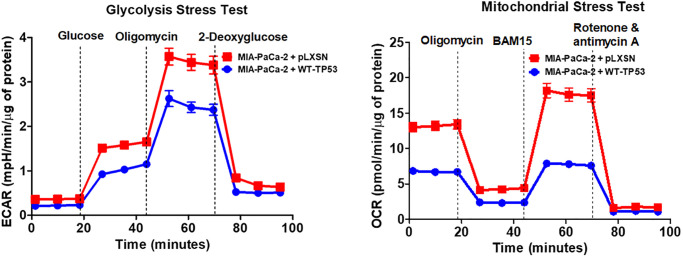
**Effects of presence of WT-TP53 on glycolysis and mitochondrial respiration.** The data for MIA-PaCa-2 + pLXSN is the same control as presented in [91]. Both MIA-PaCa-2 + pLXSN and MIA-PaCa-2 + WT-TP53 cells were examined the same time on the Seahorse machine as were MIA-PaCa-2 + WT-GSK-3β and MIA-PaCa-2 + KD-GSK-3β cells (all four cell lines done at same time). The data presented in Figure 14 are the means and standard error of the means (SEM).

**Figure 14:**
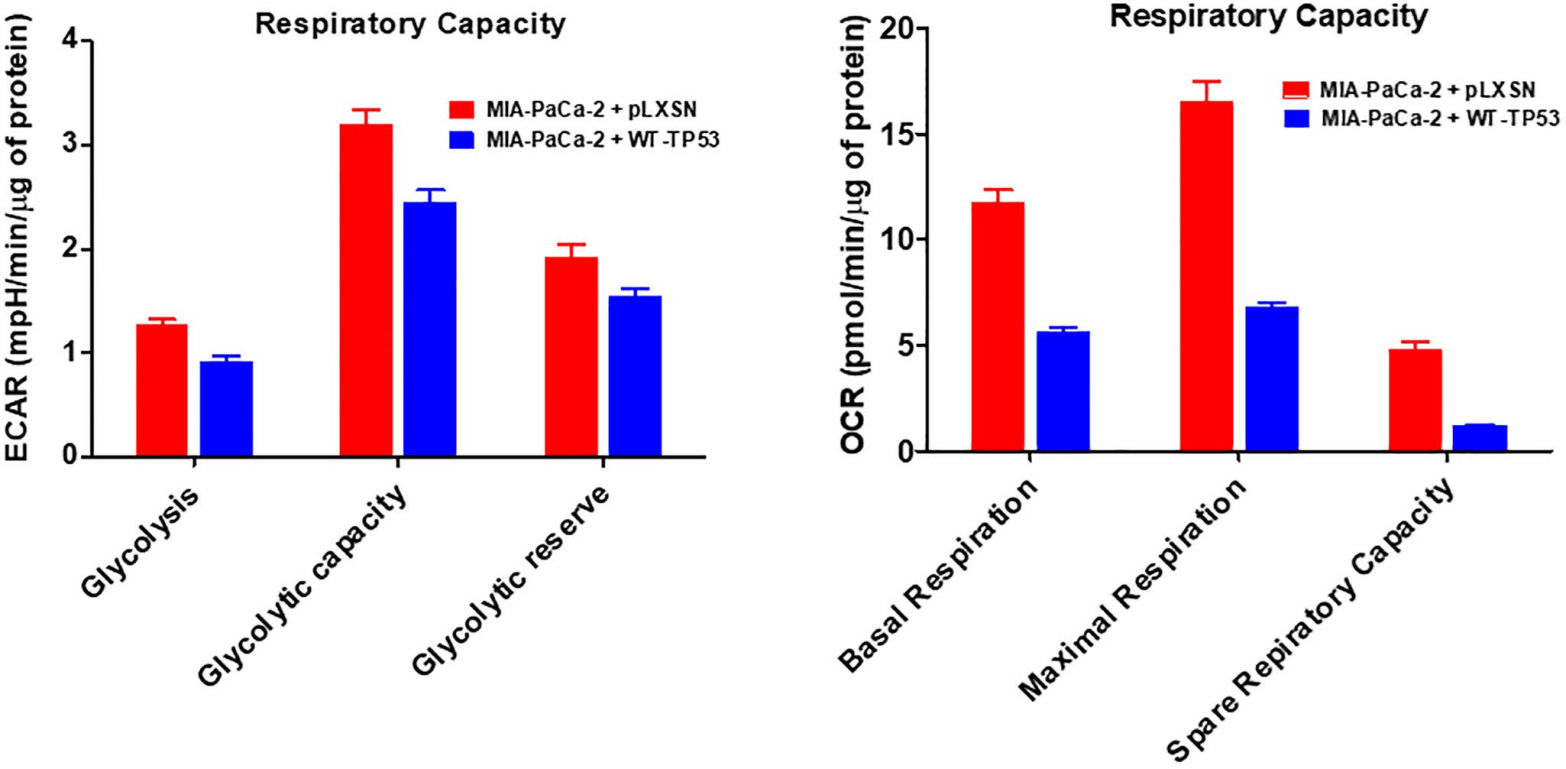
**Effects of presence of WT-TP53 on respiratory capacity.** The data for MIA-PaCa-2 + pLXSN is the same control as presented in [91]. Both MIA-PaCa-2 + pLXSN and MIA-PaCa-2 + WT-TP53 were examined the same time on the Seahorse machine. The measurements were made 5 times (5 replicates). The data presented in Figure 14 are the means and standard error of the means (SEM).

**Figure 15:**
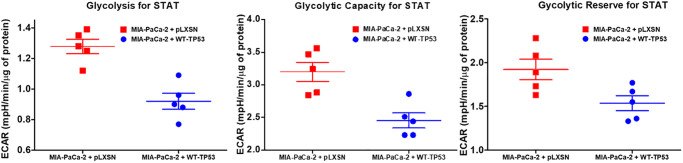
**Effects of presence of WT-TP53 on glycolysis.** Glycolysis for STAT, glycolytic capacity, and glycolytic reserve for STAT were measured by the Seahorse instrument. The data for MIA-PaCa-2 + pLXSN is the same control as presented in [91]. Both MIA-PaCa-2 + MIA-PaCa-2 + WT-TP53 were examined the same time on the Seahorse machine. STAT is an abbreviation for statistics used in study which was the Mann–Whitney test.

The effects of WT-TP53 activity on metabolic parameters were determined in MIA-PaCa-2 cells containing and lacking WT-TP53 activity were determined as we previously described [90] using the Seahorse analyzer. The results presented here indicated that restoration of WT-TP53 activity led to a decrease in glycolytic capacity in comparison to cells lacking WT-TP53 activity (Figures 13–15). Moreover, the effects on mitochondrial respiration also were more pronounced in MIA-PaCa-2 cells containing WT-TP53 activity.

Upon restoration of WT-TP53 activity in MIA-PaCa-2 cells, the level of basal mitochondrial respiration was significantly lower than in MIA-PaCa-2 lacking WT-TP53 activity. Also, their maximal respiratory and spare respiratory capacity levels were significantly reduced in contrast to cells lacking WT-TP53 (Figures 13 and 14).

## DISCUSSION

*TP53* is one of the most frequently mutated genes in human cancer, including pancreatic cancer. The *TP53* genes are altered in the two PDAC cell lines examined.

MIA-PaCa-2 cells have GOF TP53 mutations and PANC-28 cells lack TP53 expression. Both PDAC cell lines have activating mutations in the *KRAS* gene which results in constitutive KRas expression. Interactions between mutant TP53 and KRas have been observed which led to increased KRas functions [51]. GSK-3β may regulate KRas activity in these cells [51]. Thus, TP53 can interact with many signaling pathways important in cancer development.

In this manuscript, the consequences of restoration of WT-TP53 activity on the response to therapeutic agents have been documented. Restoration of WT-TP53 activity augmented the ability of PDAC cells to various agents used in the therapy of many different cancer types.

Interestingly, restoration of WT-TP53 activity augmented the responsive of MIA-PaCa-2 cells to multiple small molecule inhibitors which target critical signal molecules which are often aberrantly regulated in various cancers. These kinases and GTPases are often associated with cell growth and metastasis.

When WT-TP53 activity was restored to MIA-PaCa-2 cells they became more sensitive to small molecule inhibitors that target mutant KRas and downstream MEK1 than cells containing pLXSN. Thus, WT-TP53 could increase the sensitivity of cells which contain mutant KRas to MEK1 inhibitors. ERK1,2 lies downstream of MEK1. ERK1,2 phosphorylates many important substrates which are involved in various aspects of cell proliferation. Combination of ERK1,2 and autophagy inhibition with a MEK1 inhibitor and chloroquine may be an additional treatment option for some PDAC patients [91].

The presence of functional WT-TP53 is important for the sensitivity of FL5.12 hematopoietic cells to the mTORC1 blocker rapamycin [92]. FL5.12 cells normally have WT-TP53 activity [93]. Upon insertion of dominant negative (DN) TP53 into FL5.12 cells, their sensitivity to rapamycin was eliminated [92]. Likewise, in this current study, restoration of WT-TP53 activity in MIA-PaCa-2 cells increased the sensitivity to rapamycin. Thus, TP53 intersects with the mTORC1 pathway.

Clearly, the presence of WT-TP53 is critical for the sensitivity of various cancers, including PDAC to many drugs used in cancer therapy [94]. Additional studies on methods and approaches to reactivate mutant TP53 and other mutated genes implicated cancer should be undertaken.

**Figure 16:**
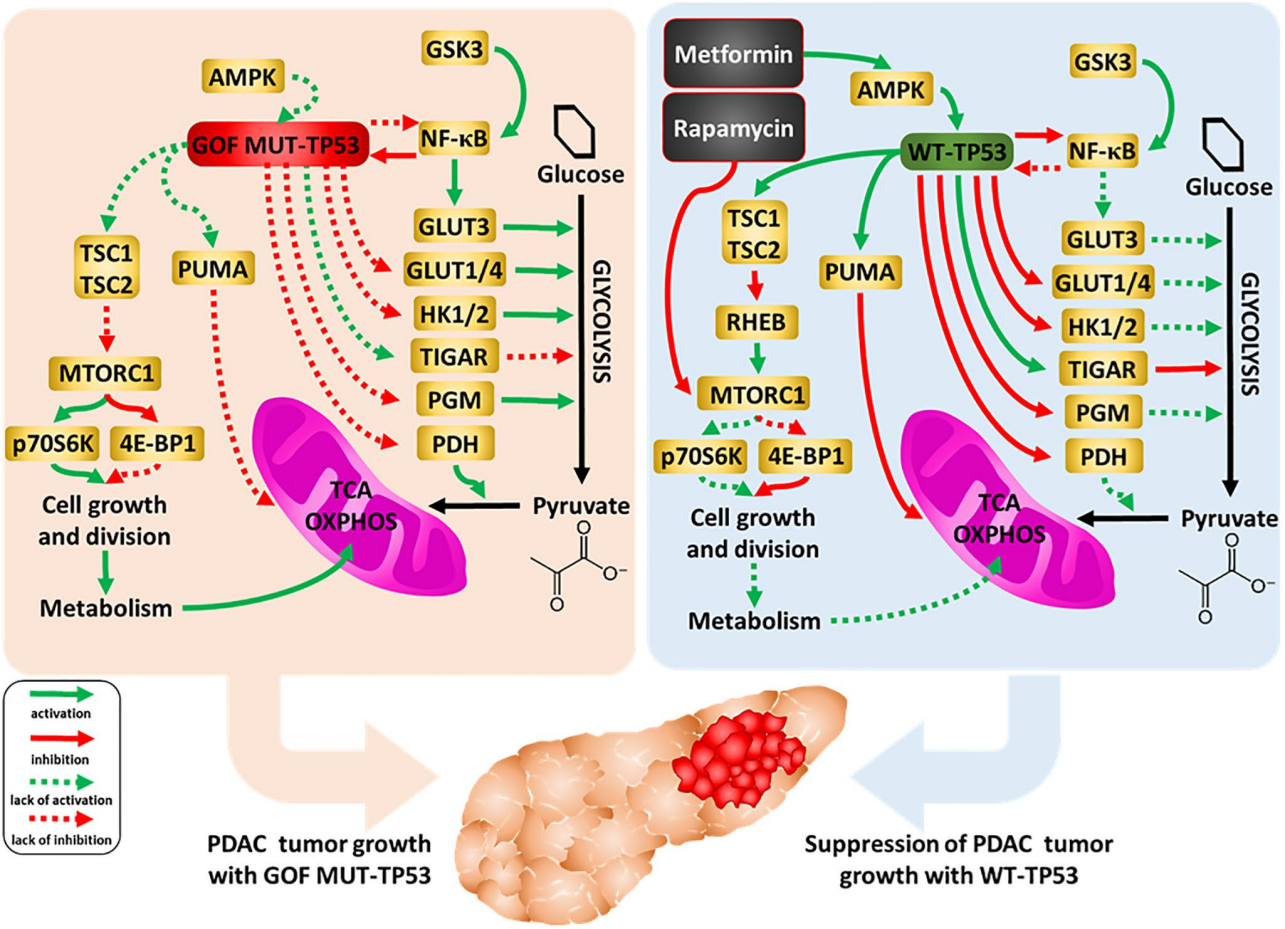
**Influences of mutant and WT-TP53 on mitochondrial activity and glucose metabolism and effects of rapamycin and metformin.** The effects of WT and mutant TP53 on key enzymes important in glycolysis and how they can influence metabolism and PDAC tumor growth. In our studies, we have examined the effect of GOF mutant TP53 and in some cases WT TP53. In addition, sites of interaction of the type 2 diabetes drug metformin and the immunosuppressive drug rapamycin and their effects on AMPK and mTORC1 are indicated. TP53 can induce mitochondrial apoptosis pathway by regulating the expression of PUMA and other proteins.

TP53 can influence glycolytic and mitochondrial metabolism both through transcriptional and non-transcriptional regulation. This influence is important for the tumor suppressor role of the protein. An overview of the effects of WT and mutant TP53 on metabolic properties, together with the effects of metformin and rapamycin, and drugs used to inhibit pancreatic cancer growth, is presented in Figure 16.

Enhanced glucose metabolism via glycolysis is the predominant source of ATP in numerous cancers. TP53 represses expression of, for example, glucose transporters, hexokinase and inhibits nuclear factor-kappa B cell (NF-κB), a protein that regulates many genes, including genes encoding glycolytic enzymes. Thus, restoration of WT TP53 activity can lead to reduction of glycolysis and impairment of cancer cell growth. On the other hand, TP53 is known to induce oxidative phosphorylation and mitochondrial production of intermediates for biosynthesis [for review see 89]. In our work, however, we observed a decrease of both glycolysis and mitochondrial respiration after the restoration of WT TP53 activity in PDAC. Other studies with breast cancer cell lines, which differ in TP53 status as well as other genes, were observed to increase both glycolytic and mitochondrial activity when mutant TP53 was present [95]. Knock-in of certain TP53 GOF mutations in mice was observed to augment mitochondrial activity, promote survival, and increased maximal treadmill exercise times [96].

TP53 has been shown to induce pro-oxidant enzymes and mitochondrial apoptosis pathway (by regulating the expression of PUMA, BAK, BAX, BCL2, BCLXL), and block anti-oxidant pathways [88]. Thus, the observed reduction of mitochondrial respiration might result from the oxidative-stress-induced impairment of function of the organelles in the WT-TP53-expressing cells. The observed value of the maximum respiration of these cells, only slightly higher than the basal respiration, seems to confirm the impairment of mitochondrial function, but it should be kept in mind that the reduction of the glycolytic rate leads to a reduction in the number of mitochondrial substrates.

Regardless of which of the above processes contributes more to the reduction of mitochondrial metabolism in comparison with the same cells that only express GOF TP53, together the observed changes suggest restoration of WT-TP3 activity confers increased sensitization to various drugs and therapeutic molecules, natural products as well as nutraceuticals. Mutant TP53 can affect the activity of mTORC1 which is important in cellular growth and metabolism. Mutant TP53 may make the PDAC cells more resistant to rapamycin than cells containing WT-TP53. Rapamycin and metformin can interfere with some of the important pathways in the mitochondria, some of which are regulated by TP53 [96–98].

## MATERIALS AND METHODS

### Cell culture and sources of therapeutic agents

The MIA-PaCa-2 and PANC-28 cells have been described in previous publications [37, 39, 99]. Cell culture conditions and sources of chemotherapeutic drugs, small molecule inhibitors, natural products and nutraceuticals have been described in our previous publications [40, 41, 47, 80, 90, 100]. Aclacinomycin was obtained from the US National Cancer Institute, (Bethesda, Maryland, USA).

### Restoration of WT-TP53 activity

Restoration of WT-TP53 activity and sources of plasmid DNAs have been previously described [40, 41, 90].

### Cell proliferation assays-MTT assays

MTT assays were performed as described previously [40].

### Clonogenicity assays

Clonogenicity Assays were performed as described in our previous publication [100].

### Semi-solid colony formation

Semi-solid colony formation in agar has been described in our previous publications [100, 101].

### Analysis of cell metabolism

Cellular metabolism and statistical analysis were performed as described in our previous publication [90].
